# Preservation of Bone and Soft Tissue Components of the Alveolar Ridge during Immediate Implantation in the Aesthetic Zone of Jaws with Bone Deficiency

**DOI:** 10.17691/stm2020.12.1.07

**Published:** 2020

**Authors:** M.V. Dyakova, N.A. Bespalova, A.S. Klochkov, E.A. Durnovo

**Affiliations:** Tutor, Department of Surgical Dentistry and Maxillofacial Surgery with the Course of Plastic Surgery, Privolzhsky Research Medical University, 10/1 Minin and Pozharsky Square, Nizhny Novgorod, 603005, Russia; Associate Professor, Department of Surgical Dentistry and Maxillofacial Surgery with the Course of Plastic Surgery, Privolzhsky Research Medical University, 10/1 Minin and Pozharsky Square, Nizhny Novgorod, 603005, Russia; Associate Professor, Department of Surgical Dentistry and Maxillofacial Surgery with the Course of Plastic Surgery, Privolzhsky Research Medical University, 10/1 Minin and Pozharsky Square, Nizhny Novgorod, 603005, Russia; Professor, Head of the Department of Surgical Dentistry and Maxillofacial Surgery with the Course of Plastic Surgery, Privolzhsky Research Medical University, 10/1 Minin and Pozharsky Square, Nizhny Novgorod, 603005, Russia

**Keywords:** immediate implantation, gum biotype, facial alveolar bone wall, smile zone, aesthetically significant jaw area, combined connective tissue graft

## Abstract

The use of immediate implantation (directly into the extracted tooth socket) in the aesthetic zone is a rather difficult task due to the specific structural features of the bone tissue of the alveolar processes and parts of the jaw.

**The aim of the study** was to develop and evaluate the efficacy of the new method of immediate implantation during restoration of teeth in the anterior part of the jaw with bone deficiency, which allows obtaining a high aesthetic result.

**Materials and Methods:**

The clinical study involved patients with partially edentulous anterior maxillae, chronic apical periodontitis and dental root fractures in the absence of possibility to restore these teeth with orthopedic structures. To identify the main criteria determining smile aesthetics, the detailed analysis of changes in the bone and soft tissues of the alveolar processes was made based on tooth extraction causes and dates. These criteria included gum biotype, the height of the distal and mesial interdental papillae; the width of the keratinized attached gingival area, gingival zeniths, the alveolar ridge thickness. The condition of the facial alveolar bone wall in the planned intervention area was assessed and its thickness was measured using cone-beam CT scan of the jaw. These parameters were measured during traditional immediate implantation, immediate implantation with a free connective tissue graft, implantation performed using the method developed by the authors and during delayed implantation in the anterior part. In each patient, the obtained data were compared with the results in the respective teeth area on the opposite side before the surgery, 4, 6 months and 1 year after the surgery.

**Results:**

Clinical and X-ray studies of the developed method of immediate implantation in the aesthetic zone of the jaw with bone deficiency in the facial alveolar bone wall have convincingly demonstrated its efficacy in the long term (1 year after the surgery). The proposed protocol has made it possible to reduce the length of rehabilitation time, and most importantly, to stabilize and preserve the alveolar ridge architecture.

**Conclusion:**

The proposed method showed the promising outlook for dental implant-supported restoration in difficult anatomical conditions.

## Introduction

The focus of prospective scientific research in the field of modern dentistry has shifted toward addressing the issues of achieving long-term and stable results in implant treatment, especially in the anterior aesthetic segment of the jaw [1–3]. Bone modeling developing fast during the first 3–6 months post-extraction is characterized by the loss of almost 50% of bone mass and significant negative dynamics in soft tissue condition and volume. A decrease in the height of the interdental papillae, changes in the gingival contour, biotype and morphology of the edentulous crest is accompanied by developing “tension syndrome”, which further aggravates the clinical situation, especially, in the aesthetic segment of the jaw [4–6]. These changes require multi-stage preparation for implant placement (bone and soft tissue augmentation), which increases rehabilitation time and often fails to provide a successful result [7, 8].

Immediate implantation, i.e. placement of a dental implant directly into the extraction socket, has become the method of choice for most specialists [9–11]. Such treatment protocol allows preserving the volume of bone tissue, providing support for soft tissues, and reducing treatment time, which is particularly important for the anterior jaw segment visualized when smiling [12]. A beautiful natural smile depends not only on dental health, but also on the appearance of the gums.

However, the method of immediate implantation in the anterior segment is often limited by the morphological structure of the bone tissue of the alveolar processes and parts of the jaw — a thin facial wall with thickness of less than 1 mm in 75% of cases, it undergoes resorption after tooth extraction in almost 100% of cases [13]. The “bundle” bone — the internal component of the alveolar socket wall — is a tooth-dependent structure which regresses quickly after tooth extraction, leading first to horizontal and then vertical bone defects followed by changes in the gingival contour in a very short time [14]. Defects of the anterior alveolar bone wall also occur during pathological processes: inflammation, trauma resulting from endodontic teeth preparation for prosthetic repair [15]. According to some authors, dental implantation is contraindicated in such conditions as it leads to unsatisfactory aesthetic results, especially in cases of the thin gingival biotype, and the implant life is reduced [16, 17]. This determines the relevance of finding new methods of immediate implantation in complex clinical situations in the aesthetic zone of the jaw.

**The aim of the study** is to develop a protocol for immediate implantation in the anterior maxilla with bone deficiency and evaluate its efficacy.

## Materials and Methods

Our study was carried out in the clinics of the Department of Surgical Dentistry and Maxillofacial Surgery with the Course of Plastic Surgery at Privolzhsky Research Medical University. A total of 69 patients were operated on — 23 males (33%) and 46 females (67%). In accordance with the Helsinki Declaration (2013), written informed consent was obtained from every patient. The study was performed following approval by the Ethics Committee of Privolzhsky Research Medical University.

**Figure 1:**
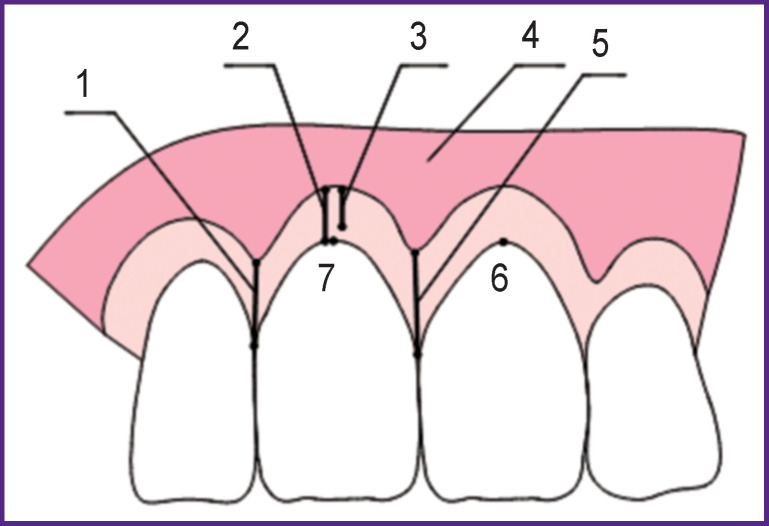
The main aesthetic parameters of soft tissues: (*1*) the height of the distal interdental papilla (mm); (*2*) the depth of the oral vestibule (mm); (*3*) keratinized (attached) gingival area (mm); (*4*) non-keratinized (free) gingival area (mm); (*5*) the height of the mesial interdental papilla (mm); (*6*) the gingival zenith of the respective tooth on the opposite side; (*7*) the gingival zenith of the examined tooth

***Clinical examination*.** Before the surgery, all patients were examined according to the developed Aesthetic Charts based on the modified Pink Aesthetic Score by Fürhauser et al. [18]. The Charts included the most important aesthetic parameters of the soft tissues: the height of the distal and mesial interdental papillae; the width of the keratinized gingival area; the gingival zenith; the thickness of the alveolar ridge as compared to the respective tooth area on the opposite side (Figure 1).

**Figure 2:**
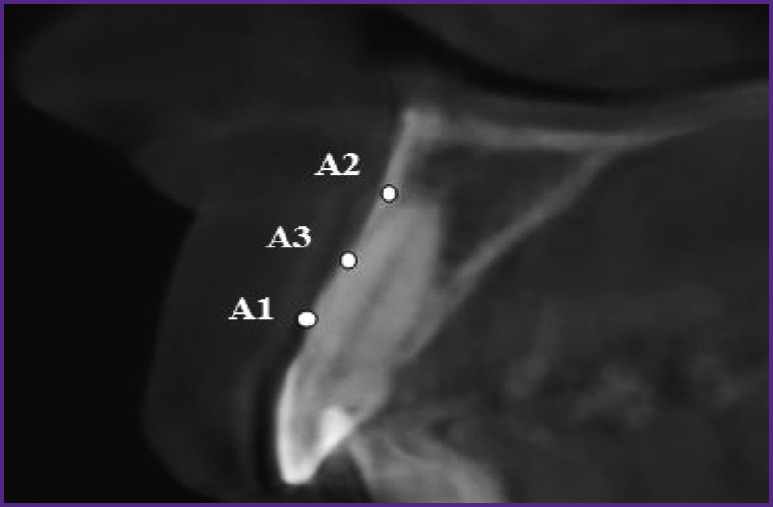
Measuring the facial alveolar bone wall thickness in the region of tooth 1.1 using cone-beam CT (mm)

***X-ray examination*.** The alveolar ridge thickness and height, as well as facial alveolar bone wall thickness, were measured in millimeters at points A1, A2, A3 in all patients using cone-beam CT (Figure 2).

At the same time, soft tissue indices (gingival radio intensity) were determined in the intervention area using the Hounsfield unit (HU) scale.

All of these parameters were measured 4, 6 months and 1 year post-surgery. Indicators obtained in the long-term postoperative period (1 year post-surgery) appeared to be particularly significant as it is the time when recessions with the development of aesthetic complications are recorded in the area of implants.

***The method of immediate implantation*.** We have proposed and patented a new method for immediate dental implantation [19]. The developed protocol makes it possible to preserve and shape the alveolar ridge contours by introducing xenogenic collagen matrix, collagen membrane, and thereby to stabilize the facial alveolar bone. The marginal gingiva is firmed and stabilized due to more densely located collagen fibers contained in the epithelial margin and the de-epithelialized zone of combined connective tissue grafts that we use to prevent the recurrence of recessions in the implant area in the long term. The area consisting of loose connective tissue is distributed over the entire surface of the middle and apical thirds of the facial bone wall, increasing the volume of tissues that protect the subjacent bone mass and the xenogenic graft [20, 21].

To study the efficacy of the proposed method, the patients were divided into three groups in compliance with the protocol of surgical treatment in the aesthetic zone of the maxilla, according to the classification developed by Elian et al. [22]. Groups 1 and 2 included patients with the diagnoses of “chronic apical periodontitis” and “tooth root fracture”, group 3 comprised patients diagnosed with “partial adentia” in the anterior.

In group 1 (n=21), the patients had medium or thick gingival biotype, the thickness of the intact facial bone wall was 1 mm or more; the treatment plan included immediate implantation according to the traditional protocol [23].

Group 2 (n=23) included patients diagnosed with defects in the upper third of the facial alveolar bone wall (5 mm or less) or the wall thickness was less than 1 mm; the thin gingival biotype was noticed. Twelve persons from this group underwent surgery using the method of immediate implantation and plastic reconstruction of the soft tissue with free connective tissue grafts (group 2a). This type of graft for soft tissue repair was chosen due to its great biological potential [24]. It definitely provides the possibility to increase tissue thickness, though its loose structure does not always allow increasing the gingival density and changing the gingival morphology in the neck area of the implant, which is of key importance in preventing recessions. Therefore, the remaining 11 patients of group 2 (group 2b) received treatment according to the developed protocol of immediate implantation.

**Figure 3:**
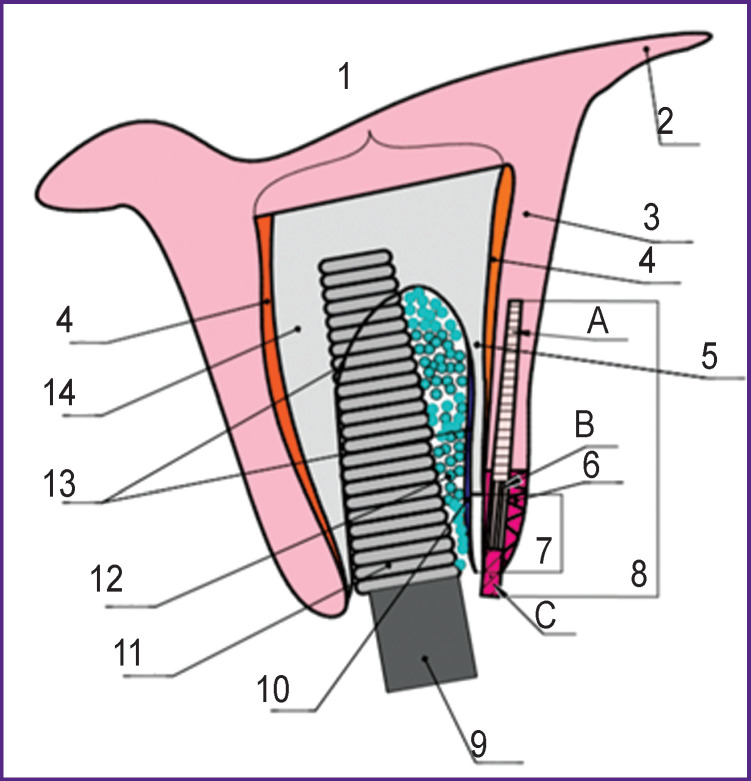
The diagram of dental implant placement in patients of group 2: (*1*) alveolar bone; (*2*) transitory fold; (*3*) non-keratinized gingiva; (*4*) periosteum; (*5*) facial alveolar bone wall; (*6*) keratinized gingiva; (*7*) a bone defect in the facial bone wall; (*8*) combined soft tissue graft: (*A*) connective tissue zone; (*B*) de-epithelized zone; (*C*) epithelial margin (1.5 mm); (*9*) healing cap; (*10*) collagen membrane; (*11*) dental implant; (*12*) osteoplastic material; (*13*) the boundaries of the extraction socket; (*14*) the palatal wall of the alveolar bone

This approach is distinguished by the use of a combined autograft containing the de-epithelized zone and the epithelial margin in addition to the connective tissue. This type of tissue has more densely arranged collagen fibers, which allows increasing the gingival density and thereby stabilizing the gingival margin and the subjacent xenogenic material, the anterior alveolar wall (Figure 3).

Thus, we solved several problems in one: reducing the treatment time by immediate placement of the dental implant into the tooth socket, preserving the interdental papillae and zenith of the gingival contour, altering the soft tissue biotype, fixing the facial bone wall, which helped to increase predictability and achieve high esthetic results of implant treatment.

Group 3 (n=25) included patients with bone and soft tissue defects formed in the anterior maxilla after tooth extraction, which prevented placement of dental implants. They underwent delayed implantation with bone grafting and soft tissue augmentation in several stages [25].

***Statistics*.** Processing of the obtained data was carried out by generally accepted statistical methods using the Statistica 6.0 software package (StatSoft Inc., USA). To evaluate the efficacy of the proposed treatment method, calculation was performed in absolute numbers (mm) and percent (%). Student t-test and Fisher tables were used to determine reliability of the research results for small samples. The differences were considered statistically significant at p≤0.05.

## Results and Discussion

The results of this study suggest the efficacy of the proposed method of immediate implantation in obtaining the most stable gingival contour, preserving the alveolar ridge volume in the dental implant area of the smile zone (Table 1).

**Table 1:** Dynamics of aesthetic indices 1 year after dental implantation (M±m)

Indices	Group 1			Group 2a			Group 2b			Group 3		
	Mean value (mm)	Difference from the value before surgery (%)	Difference from RTOOS value (%)	Mean value (mm)	Difference from the value before surgery (%)	Difference from RTOOS value (%)	Mean value (mm)	Difference from the value before surgery (%)	Difference from RTOOS value (%)	Mean value (mm)	Difference from the value before surgery (%)	Difference from RTOOS value (%)
The depth of the oral vestibule	7.90±1.52	–0.63	–3.89	4.13±0.71	6.17	–10.80	5.81±1.89	–11.30	–14.10	6.32±0.44	64.58*	–7.06
KAGAW	5.93±1.93	–0.67	–8.91	4.33±0.23	–3.13	–5.46	6.40±1.02	–3.03	–10.74	4.52±0.07	92.34*	6.60
The difference between the gingival zeniths	0.21±0.04	162.50*	—	0.05±0.02	–76.19*	—	0.10±0.02	–91.67*	—	0.20±0.71	–87.01*	—
Alveolar ridge thickness with account of the mucous membrane	7.56±0.80	–12.70	–12.73*	8.78±0.76	3.54	–3.30	8.83±1.22	7.65	4.02	8.26±0.54	–3.28	–97.08*
The height of the mesial interdental papilla	2.61±0.30	–1.15	–6.52	2.33±0.61	–0.85	–4.12	2.60±0.16	–2.26	–2.25	1.48±0.64	–15.43	–29.86*
The height of the distal interdental papilla	2.21±0.62	–3.07	–3.49	2.28±0.59	–2.41	–3.39	2.60±0.12	–2.62	–2.26	1.53±0.75	–0.64	–36.78*
Facial bone wall thickness according to cone-beam CT after 6 months:												
A1	0.73±0.17	–59.84*	—	0.27±0.05	–40.0*	—	0.22±0.05	–52.18*	—	—	—	—
A2	0.80±0.45	–66.12*	—	0.26±0.02	–58.70*	—	0.21±0.04	–58.0*	—	—	—	—
A3	0.82±0.22	–58.16*	—	0.45±0.05	–48.30*	—	0.31±0.04	–61.73*	—	—	—	—

* Statistically significant difference in indices, p≤0.05. Here: KAGAW — keratinized attached gingival area width; RTOOS — the respective tooth on the opposite side.

**Figure 4:**
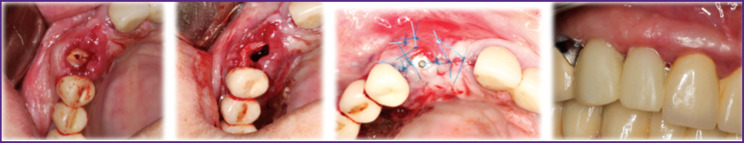
Patient Z., 52 years old. Immediate implantation of tooth 1.3 according to the developed surgical protocol and subsequent orthopedic restoration

Significant positive changes in the “pink aesthetics” were observed in groups where combined and connective tissue grafts were used (2a and 2b) as compared to group 1 where immediate implantation was performed according to the traditional protocol (Figure 4).

**Figure 5:**
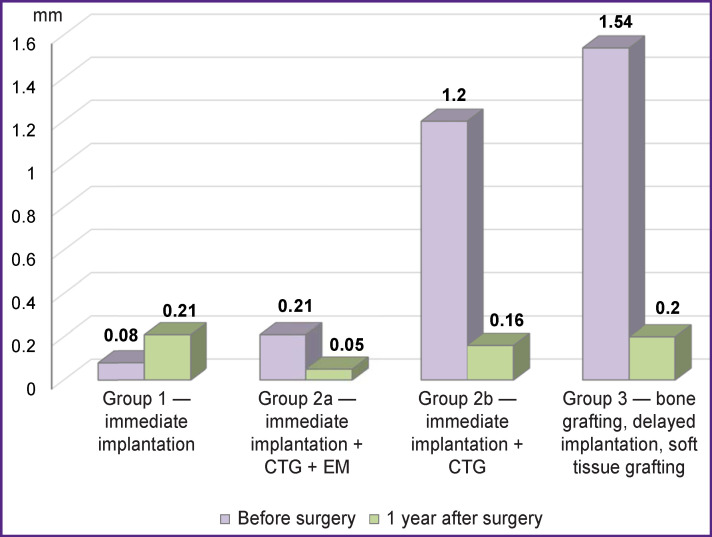
The difference between the gingival zeniths 1 year after the surgery Here: CTG — connective tissue graft; EM — epithelial margin

When using a connective tissue graft, the difference between the gingival zeniths of this and the respective tooth on the other side was reduced by 76% and in case of the combined graft by 92% (Figure 5).

One of the most important prognostic criteria for long-term implant survival is the width of the keratinized attached gingival area. Precisely this area becomes a powerful barrier protecting the underlying bone from bacterial invasion and further resorption [26]. In our study, this parameter was stable in groups 1 and 2: it actually remained unchanged as compared to the values before the surgery and was on par with the same parameter of the respective tooth on the opposite side (p>0.05). This confirms once again that immediate implantation allows preserving the soft tissue architecture and the use of soft tissue grafts helps preserve the interdental papillae, preventing formation of black triangles and providing high aesthetic results [27–29].

In groups 1 and 2, the decrease in the facial bone wall thickness was more than 50% as compared to the values before the surgery, which confirms the data provided in studies [4–6]. A statistically significant decrease in the thickness of the alveolar ridge by 58–66% (p≤0.05) was achieved during immediate implantation according to the classical protocol, which was associated with partial resorption of the facial bone wall. In group 1, an increase in the difference between the gingival zeniths by 163% 1 year after the surgery indicated the absence of positive aesthetic results and unfavorable treatment outcomes (p≤0.05).

In contrast to group 1, the use of soft tissue grafts for immediate implantation allowed changing the soft tissue biotype, increasing the gingival thickness by 68% (p≤0.05), preserving and correcting the zenith of the gingival margin (Table 2).

**Table 2:** Dynamics of the mucous membrane thickness in the facial bone area 1 year after dental implantation (M±m)

Indices	Group 1	Group 2a		Group 2b		Group 3	
		The thin biotype	The medium biotype	The thin biotype	The medium biotype	The thin biotype	The medium biotype
1 year after surgery (mm)	1.71±0.45	1.51±0.30*	1.91±0.10*	1.25±0.13*	2.10±0.50*	1.53±0.10*	1.52±0.22
Difference from the value before surgery (%)	–2.84	86.42*	55.74*	47.06*	68.0*	86.59*	14.29
Difference from RTOOS value (%)	–1.18	84.15*	55.74*	47.0*	40.48*	10.10	5.72

* Statistically significant difference in indices, p≤0.05. Here: RTOOS — the respective tooth on the opposite side.

In group 3, bone grafting in the presence of pronounced defects led to a significant increase in bone mass by more than 104% (p≤0.05) and subsequent use of free gingival grafts increased the width of the keratinized gingival area and deepened the oral vestibule by 60–90% from the initial values. However, multiple operations leading to cicatricial deformities, the absence of interdental gingival papillae (the deficit amounted to 15% compared to the respective tooth area on the opposite side), longterm rehabilitation (11.5 months on average) limited the indications for delayed implantation in the anterior in favor of the developed immediate protocol.

In comparison with the delayed multi-stage protocol, the method of immediate implantation can also reduce rehabilitation time almost by half, which is definitely an advantage in conditions of increased demands of modern dental patients.

## Conclusion

The developed novel method of immediate implantation in conditions of bone deficiency provides objectively and reliably high aesthetic results of implant treatment and stable rehabilitation in partially edentulous patients. Long-term results achieved when using the method in conditions of complex dentoalveolar morphology, i.e. a thin facial alveolar bone wall (1 mm or less), its defects and the thin gingival biotype, are particularly valuable.

The definite advantage of the proposed method is reduction of rehabilitation time for patients almost by half as compared to traditional two-stage implant treatment methods.

## References

[1] Bondarenko N.A., Losev F.F., Bondarenko A.N (2010). The necessity in dental implantation and frequency of its application.. Kubanskiy nauchnyy meditsinskiy vestnik.

[2] Gus’kov A.V., Mitin N.E., Zimankov D.A., Mirnigmatova D.B., Grishin M.I. (2017). Dental implants: state of the question today (literature review).. Klinicheskaya stomatologiya.

[3] Schwartz-Arad D. (2017). Esthetic in dentistry..

[4] Araujo M.G., Lindhe J (2005). Dimensional ridge alterations following tooth extraction. An experimental study in the dog.. J Clin Periodontol.

[5] Araújo M., Lindhe J. (2008). The edentulous alveolar ridge.. Clinical periodontology and implant dentistry..

[6] Chappius V., Araújo M.G., Buser D (2017). Clinical relevance of dimensional bone and soft tissue alterations post-extraction in esthetic sites.. Periodontol 2000.

[7] Hämmerle C.H.F., Tarnow D. (2018). The etiology of hard- and soft-tissue deficiencies at dental implants: a narrative review.. J Periodontol.

[8] Muraev A.A., Gazhva Y.V., Ivashkevich S.G., Riabova V.M., Korotkova N.L., Semyonova Y.A., Metsuku I.N., Faizullin R.L., Ivanov S.Y (2017). A novel approach to alveolar bone complex defects 3D reconstruction.. Sovremennye tehnologii v medicine.

[9] Buser D., Chappuis V., Belser U.C., Chen S (2017). Implant placement post extraction in esthetic single tooth sites: when immediate, when early, when late?. Periodontol 2000.

[10] Yaremenko A.I., Kotenko M.V., Razdorsky V.V., Snezhko V.V (2012). Analysis of the prosthetics results using the method of the immediate implantation (part 1).. Institut stomatologii.

[11] Yaremenko A.I., Kotenko M.V., Razdorsky V.V., Snezhko V.V (2013). Analysis of the prosthetics results using the method of the immediate implantation (part 2).. Institut stomatologii.

[12] Polupan P.V (2014). One-stage implantation is a new horizon in implantology.. Dental Tribune. Russian Edition.

[13] Januário A.L., Duarte W.R., Barriviera M., Mesti J.C., Araújo M.G., Lindhe J. (2011). Dimension of the facial bone wall in the anterior maxilla: a cone-beam computed tomography study.. Clin Oral Implants Res.

[14] Van der Weijden F., Dell’Acqua F., Slot D.E. (2009). Alveolar bone dimensional changes of post-extraction sockets in humans: a systematic review.. J Clin Periodontol.

[15] Durnovo E.A., Klochkov A.S., Kazakov A.V (2013). Immediate implantation after extraction of teeth with chronic apical periodontitis.. Stomatologiya.

[16] Qahash M., Susin C., Polimeni G., Hall J., Wikesjö U.M. (2008). Bone healing dynamics at buccal peri-implant sites.. Clin Oral Implants Res.

[17] Roe P., Kan J.Y., Rungcharassaeng K., Caruso J.M., Zimmerman G., Mesquida J (2012). Horizontal and vertical dimensional changes of peri-implant facial bone following immediate placement and provisionalization of maxillary anterior single implants: a 1-year cone beam computed tomography study.. Int J Oral Maxillofac Implants.

[18] Fürhauser R., Florescu D., Benesch T., Haas R., Mailath G., Watzek G. (2005). Evaluation of soft tissue around single-tooth implant crowns: the pink esthetic score.. Clin Oral Implants Res.

[19] Durnovo E.A., Andreeva M.V., Bespalova N.A., Yanova N.A. (2017). Method of direct dental implantation..

[20] Zucchelli G., Sharma P., Mounssif I (2018). Esthetics in periodontics and implantology.. Periodontol 2000.

[21] Bespalova N.A., Yanova N.A., Runova N.B., Durnovo E.A (2016). The achievement of soft tissue stability around the teeth and implants.. Rossiyskiy vestnik dental’noy implantologii.

[22] Elian N., Cho S.C., Froum S., Smith R.B., Tarnow D.P. (2007). A simplified socket classification and repair technique.. Pract Proced Aesthet Dent.

[23] Schwartz-Arad D., Laviv A., Levin L. (2007). Survival of immediately provisionalized dental implants placed immediately into fresh extraction sockets.. J Periodontol.

[24] Durnovo E.A., Bespalova N.A., Yanova N.A., Dyakova M.V., Korsakova A.I (2017). Resources of the soft tissue plastic surgery in the oral cavity for the prevention of peri-implantitis.. Rossiyskiy vestnik dental’noy implantologii.

[25] Brånemark P.I. (1993). Osseointegration and its experimental background.. J Prosthet Dent.

[26] Migliorati M., Amorfini L., Signori A., Biavati A.S., Benedicenti S (2015). Clinical and aesthetic outcome with post-extractive implants with or without soft tissue augmentation: a 2-year randomized clinical trial.. Clin Implant Dent Relat Res.

[27] Lutskaya I.K., Zinovenko O.G., Ivanov M.S., Nazarov I.E (2015). Immediate dental implantation after removal of premolar with a vertical fracture of the tooth root.. Sovremennaya stomatologiya.

[28] Zucchelli D. (2014). Plasticheskaya khirurgiya myagkikh tkaney polosti rta.

[29] Fevraleva A.Yu., Davidyan A.L. (2008). Atlas plasticheskoy khirurgii myagkikh tkaney vokrug implantatov.

